# Bazi Bushen Capsule Alleviates Post-Menopausal Atherosclerosis via GPER1-Dependent Anti-Inflammatory and Anti-Apoptotic Effects

**DOI:** 10.3389/fphar.2021.658998

**Published:** 2021-06-25

**Authors:** Dan Huang, Xindong Wang, Yunhong Zhu, Juexiao Gong, Junqing Liang, Yanfei Song, Yiyan Zhang, Linsheng Liu, Cong Wei

**Affiliations:** ^1^ Affiliated Hospital of Integrated Traditional Chinese and Western Medicine, Nanjing University of Chinese Medicine, Nanjing, China; ^2^ Jiangsu Province Academy of Traditional Chinese Medicine, Nanjing, China; ^3^ National Key Laboratory of Collateral Disease Research and Innovative Chinese Medicine, Shijiazhuang, China; ^4^ Department of Clinical Pharmacology, The First Affiliated Hospital of Soochow University, Suzhou, China

**Keywords:** atherosclerosis, bazi bushen capsule, lipid profile, inflammation, apoptosis, GPER1 antagonist

## Abstract

*Bazi Bushen capsule* (BZBS), as a Chinese medicine used to relieve fatigue, has been proven effective for the treatment of atherogenesis through antilipid effects. To investigate the potential mechanism of BZBS in the anti-atherosclerotic effect, Ovx/ApoE^-/-^ mice were applied to investigate the anti-atherosclerotic efficiency and potential mechanism of BZBS. Therapeutic effect was evaluated based on the number of CD68^+^ and CD3^+^ cells, the level of intercellular adhesion molecule-1 (ICAM-1) and vascular cell adhesion molecule-1 (VCAM-1), and the ratio of cleaved caspase-3/caspase-3, as well as increasing ratio of Bcl2/Bax. Human umbilical vein endothelial cells (HUVECs) were chosen to evaluate the role of GPER1. Treatment with BZBS reduced lipid deposition by reducing the numbers of CD68^+^ and CD3^+^ cells, the level of ICAM-1 and VCAM-1, and the ratio of cleaved caspase-3/caspase-3, and increasing the ratio of Bcl2/Bax as compared with the control group. In si-GPER1-treated HUVECs, the anti-apoptotic effect of BZBS was decreased. This study revealed that BZBS exhibited a clear effect against atherogenesis *via* GPER1-dependent anti-inflammatory and anti-apoptotic mechanisms. We believe that this manuscript is informative and useful for researchers pursuing the related alleviation of post-menopausal AS *via* anti-inflammatory and anti-apoptotic mechanisms.

## Introduction

Atherosclerosis (AS) is the basis of cardiovascular diseases (Gaziano et al., 2010; Libby et al., 2011), and cardiovascular diseases are the leading cause of death in women worldwide. Endogenous estrogen has a protective effect against AS in pre-menopausal women. However, in post-menopausal (PM) women, due to the lack of estrogen, the morbidity and mortality associated with AS are greatly increased (Barton, 2013). Despite strong vasculoprotective effects, exogenous estrogen has many side effects such as thrombosis and tumorigenesis (Schenck-Gustafsson et al., 2011), and clinical treatment of women with estrogen replacement therapy may prevent subclinical AS but has no benefit on cardiovascular disease risk after menopause and later in life (Mcgowan and pottern, 2000; Schenck-Gustafsson et al., 2011). Therefore, estrogen supplementation in PM women for primary and secondary prevention of cardiovascular disease is controversial.

Phytoestrogens (PEs) are a class of non-steroidal compounds that naturally occur in plants, fruits, and vegetables. PEs have structures and molecular weights similar to those of endogenous estrogen, and have estrogen-like effects without estrogen-like side effects (Poluzzi et al., 2014). Many traditional Chinese medicines contain PEs (Zhao et al., 2017; Liang et al., 2019; Yang et al., 2019), which provides new options for PM women looking for safe and effective anti-atherosclerotic drugs. *Bazi Bushen capsule* (BZBS; an eight-seed kidney-tonifying capsule) was used to relieve fatigue with a compound formula composed of 16 kinds of traditional Chinese medicines (Table 1) BZBS has been proven to improves lipid metabolism (Huang et al., 2020). Fingerprint analysis of BZBS revealed that it contained a variety of PEs, including flavones, coumarins, lignans, and the terpenoid catalpol (Huang et al., 2020).

**TABLE 1:** The ratio of the plants present in the preparation of BZBS.

Plants	Amount in application (ratio)
*Cuscuta chinensis* Lam. [Convolvulaceae; Cuscutae semen]	250
*Lycium barbarum* L. [Solanaceae; Lycii cortex]	138
*Schisandra chinensis* (Turcz.) Baill. [Schisandraceae; Schisandrae chinensis fructus]	46
*Cnidium monnieri* (L.) Cusson [Apiaceae; Cnidii fructus]	35
*Rosa laevigata* Michx. [Rosaceae; Rosae laevigatae fructus]	35
*Rubus chingii* Hu [Rosaceae; Rubi fructus]	35
*Allium tuberosum* Rottler ex Spreng. [Amaryllidaceae; Allii tuberosi semen]	35
*Toosendan fructus* [Meliaceae; Fuctus Toosendan]	23
*Epimedium brevicornu* Maxim. [ Berberidaceae; Epimedii folium]	70
*Morindae officinalis* radix [Rubiaceae; Radix Morindae Offcinalis]	35
*Cistanche deserticola* Ma [Orobanchaceae; Cistanches herba]	35
*Rehmannia root* [Orobanchaceae; Radix Rehmanniae Recens]	46
*Cyathula officinalis* K.C.Kuan [Amaranthaceae; Cyathulae radix]	35
*Panax ginseng* C.A.Mey. [Araliaceae; Ginseng radix et rhizoma]	25
*Cervus nippon* Temminck [Cervidae; Cervi Cornu Pantotrichum]	16
*Hippocampus Kelloggi* [Syngnathidae; Hippocampus]	21

Estrogen and PEs perform various physiological functions by binding to the corresponding estrogen receptors (Prossnitz and Arterburn, 2015; Rexrode, 2018). The classical estrogen nuclear receptors ERα and ERβ mediate genomic effects by regulating the transcription of specific target genes (Meyer et al., 2011). As a membrane estrogen receptor, the G protein-coupled oestrogen receptor 1 (GPER1) has a rapid nongenomic effect mediated by the second messenger system (Revankar et al., 2005; Meyer et al., 2011). The pathogenesis of AS is complex. Endothelial cells (ECs) inflammation and inflammation-induced endothelial apoptosis are triggers for the occurrence and development of AS (Madhur et al., 2011; Herrero-Fernandez et al., 2019). Moreover, the PM state is characterized by generalized inflammatory activation. Estrogen exerts potent vasculoprotective effects that are partly mediated by inhibiting inflammation and apoptosis (Barton, 2013). GPER1 is expressed in many organs of the body including the arteries (Reslan et al., 2013). In PM mice, GPER1 deficiency exacerbates AS, an effect that is associated with significant vascular inflammation. Administration of G1, a highly selective GPER1 agonist, led to a pronounced decrease in vascular inflammation accompanied by attenuated atherosclerotic plaque formation (Meyer et al., 2014).

In the current study, we aimed to investigate the potential mechanism of BZBS on AS. We investigated the effects of BZBS in an animal model of PM AS ovariectomized (Ovx) ApoE^-/-^ mice. After demonstrating that BZBS has anti-inflammatory and anti-apoptotic effects, we performed an *in vitro* study to assess the underlying mechanism. Our results revealed that these effects are dependent on the GPER1-mediated inhibition of anti-inflammatory and anti-apoptotic mechanisms.

## Materials and Methods

### Chemicals and Reagents

BZBS capsules were provided by Shijiazhuang Yiling Pharmaceutical Co., Ltd. (Lot: A1801001, Shijiazhuang, China). A BZBS suspension in 0.5% sodium carboxymethylcellulose (CMC) was administered to mice *via* intragastric injection. BZBS was weighed and dissolved in serum-free Ham’s F-12K medium. G1 was provided by ApexBio (Houston, TX, United States). As previously described, G1 was dissolved in absolute ethanol to prepare a stock solution of 20 μg/ml and stored in a -20°C freezer. The G1 working solution was diluted 10 times with solvent, and each mouse was injected subcutaneously with 0.2 μg G1 per day (Dennis et al., 2009). The high-fat diet was purchased from Research Diets, Inc. (PE-free, 1.25% cholesterol, 40 kcal% fat, and 0.5% cholic acid, New Brunswick, NJ, United States).

### Animal Experiments and Sample Preparation

Fifty C57BL/6J mice and sixty female homozygous ApoE^-/-^ mice (18–22 g) were obtained from GemPharmatech Co., Ltd. (Nanjing, China). The animals were acclimated for three-days with a 12 h light-dark cycle with water and food *ad libitum*. The experiments were approved by the Ethics Commission of Animal Care of the Hebei Yiling Chinese Medicine Research Institute under approval number N2018050 (20180711).

Surgical menopause (Ovx/ApoE^-/-^ mice) were induced by ovary ligation and bilateral ovariectomy under sterile conditions, whereas the C57BL/6J mice (control group, *n* = 15) only underwent sham surgery (needle threading). After recovering for 7 days, the ApoE^-/-^ mice were randomly assigned to four groups (*n* = 15) as described in Table 2. The first group served as the model group, and these animals received a high-fat diet with intragastric (i.g.) injection of the vehicle (0.5% CMC-Na). In the three experimental groups, the G1 group was treated with 0.2 μg/day G1 and high-fat diet, while the low-dose BZBS (1.4 g/kg/day, LD-BZ) group and the high-dose BZBS (2.8 g/kg/day, HD-BZ) groups were treated with BZBS and high-fat diet for 12 weeks.

**TABLE 2:** Treatment protocol of mice.

Treatment Regimen	Mice	Diet	Dosage in mouse (administration route)	Treatment Days
Control	C57BL/6J	Normal	0.5% CMC-Na (ig)	12 weeks
Model	ApoE^-/-^	High-fat	0.5% CMC-Na (ig)	
G1	ApoE^-/-^	High-fat	0.2 μg/d G1 (sc)	
HD-BZ	ApoE^-/-^	High-fat	2.8 g/kg/d (ig)	
LD-BZ	ApoE^-/-^	High-fat	1.4 g/kg/d (ig)	

### Cell Experiments

Human umbilical vein endothelial cells (HUVECs) were cultured in Ham’s F-12K medium supplemented with 100 μg/ml heparin, 50 μg/ml endothelial cell growth supplement (ECGS), 10% foetal bovine serum (FBS), and 1% penicillin-streptomycin (P/S) (Procell Life Science & Technology Co., Ltd., Wuhan, China) at 37°C in 5% CO_2_. Cells at passages 3–7 were used in subsequent experiments. HUVECs were seeded at a density of 1 × 10^5^ cells per well into 35-mm dishes. After 24 h, the cells were transfected with 50 nM *GPER1* siRNA (CCG​ACC​UGU​ACU​UCA​UCA​ATT and UUG​AUG​AAG​UAC​AGG​UCG​GTT; GenePharma, Shanghai, China) in Gibco Opti-MEM I reduced serum medium with Invitrogen Lipofectamine RNAiMAX transfection reagent. Scrambled siRNA without sequence homology to any known human gene served as the negative control (NC). GPER1 protein levels were measured 40 h post-transfection to evaluate the knockdown efficiency. To establish an oxidized low-density lipoprotein (Ox-LDL)-induced apoptosis model and assess the protective effect of BZBS, we pretreated transfected HUVECs with 400 μg/ml BZBS in serum-free EC basal medium. The pretreatment started 8 h after transfection, lasted for 12 h and was followed by incubation with Ox-LDL (120 μg/ml, 24 h).

### Serum Analysis

The levels of intercellular adhesion molecule-1 (ICAM-1) and vascular cell adhesion molecule-1 (VCAM-1) were quantified by an enzyme-linked immunosorbent assay (ELISA) kit (CUSABIO, Wuhan, China).

### Quantification of Atherosclerotic Lesions

For en face quantification of AS, part of the aorta from the descending aorta to the iliac bifurcation was excised, carefully cleaned to remove adherent fat and connective tissue, and mounted en face with oil red O staining solution. To observe atherosclerotic plaques under a microscope, the hearts and proximal aortae were removed, and cross-sections (8 µm serial sections) of the aortic root were stained with haematoxylin-eosin (H&E) and oil red O. Neutral lipids were stained with oil red O. Four cross-sections were analysed from each mouse. Quantification was performed using Image-Pro Plus 6.0 software (Meyer Instruments, Houston, TX, United States).

### TUNEL Apoptosis Assay

TUNEL (terminal deoxynucleotidyl transferase dUTP nick end labelling) apoptosis assays were performed with the *In Situ* Cell Death Detection Kit, Fluorescein (Roche, Mannheim, Germany). Cells grow on cover slips were fixed by 4% paraformaldehyde. Proteinase K working solution was then applied to cover the cells and incubated at 37°C for 25 min. Permeabilization working solution was added to the cover slips, and incubated at room temperature for 20 min. TDT enzyme, dUTP and buffer from the TUNEL kit were mixed at the ratio of 1:5:50 according to the number of slices. After 3% BSA blocking, rabbit polyclonal antibody to CD31 (1:100; Abcam) and goat anti-rabbit IgG (1:100; Abcam, red fluorescence) were used to confirm ECs. The cells were then incubated with a DAPI solution at room temperature for 10 min. Images were obtained using a ZEISS laser scanning confocal microscope.

### Immunofluorescence, Quantitative Real-Time PCR analysis, Western Blot Analysis, and Annexin V-FITC/Propidium Iodide Apoptosis Assay

The complete details of immunofluorescence, quantitative real-time PCR analysis, Western blot analysis, and Annexin V-FITC/propidium iodide (PI) apoptosis assay are provided in the Supplementary Material.

### Statistical Analysis

Correlation analysis was performed using IBM SPSS 20.0 software. Statistical analysis was performed by one-way ANOVA. Differences with *p*-values < 0.05 were considered statistically significant. All results are presented as mean ± SE.

## Results

### Bazi Bushen Capsule Treatment Hindered the Development of AS in Ovx/ApoE^-/-^ Mice

**FIGURE 1:**
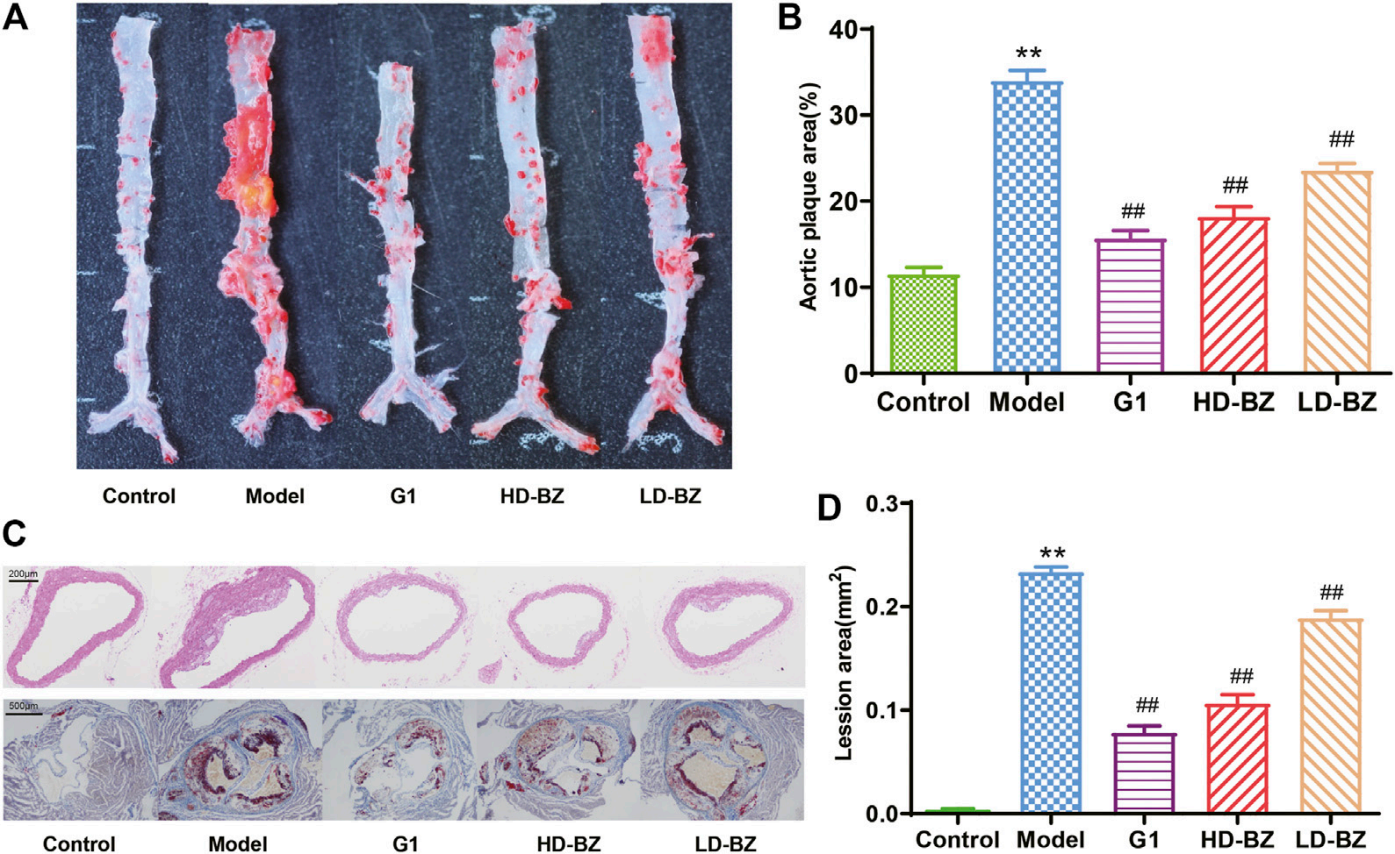
Effects of BZBS on atherosclerotic plaque formation in Ovx/ApoE^-/-^ mice. **(A)**En face staining of aortas with oil red O. **(B)** Quantification of the plaque area as measured in A. **(C)** Light microscopy images (40×) of aortic roots stained with H&E and oil red O. **(D)** Quantification of the lesion areas as measured in C. **p < 0.01 vs. the control group; ^##^ *p* < 0.01 vs. the model group (*n* = 4–6).

Both the macroscopic observation of the aortas (Figures 1A,B) and the microscopic images of the aortic roots (Figures 1C,D) revealed that lipid deposition was most severe in the model group. Treatment with G1 or BZBS reduced lipid deposition, as the lesions in the corresponding groups (G1, HD-BZ, and LD-BZ) were narrower than those of the model group (Figures 1A–D). En face analysis of the aortas revealed 29, 26, and 17% reduction in plaque area in the G1, HD-BZ, and LD-BZ groups, respectively, compared with that of the model group (Figures 1A,B), indicating that BZBS has a therapeutic effect against AS induced by estrogen deficiency and a high fat diet. These results support the idea that BZBS exhibits a protective effect against AS.

### Bazi Bushen Capsule Treatment Inhibited the Accumulation of Inflammatory Cells in Ovx/ApoE^-/-^ Mice

**FIGURE 2:**
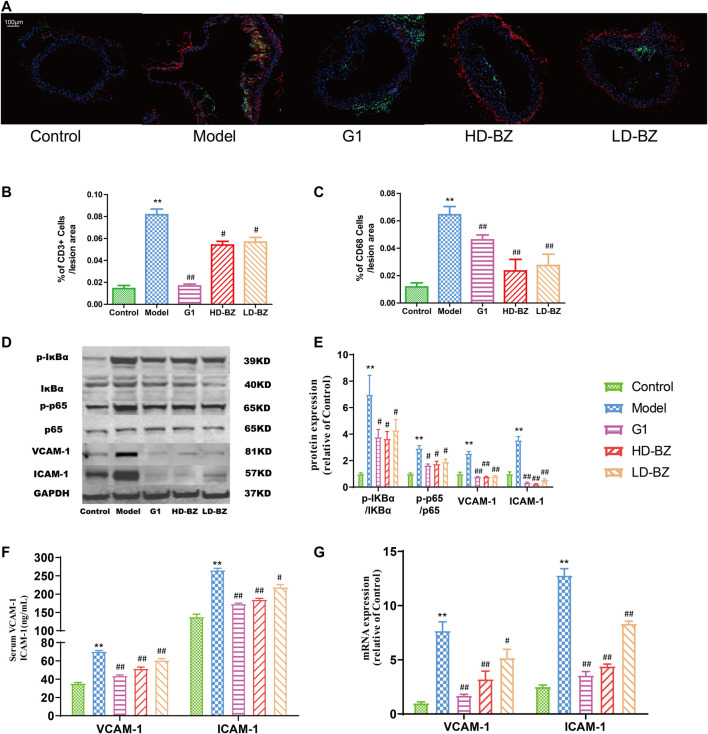
Effect of BZBS on the accumulation of inflammatory cells and adhesion molecule expression mediated by transactivation of NF-κB signalling in Ovx/ApoE^-^/-mice. **(A)** Immunofluorescence was used to quantify CD68^+^ cells (macrophages, green) and CD3^+^ cells (T cells, red) in the aortic arch. **(B)** %of CD3^+^ cells/lesion area. **(C)** %of CD68^+^ cells/lesion area. **(D)** Western blots of phospho-IκBα (Ser36), IκBα, phospho-p65, p65, VCAM-1, ICAM-1, and GAPDH. **(E)** Graph showing quantification results of the bands in D, using GAPDH as an internal reference protein. **(F)** Graph showing the levels of VCAM-1 and ICAM-1 as determined by ELISA. **(G)** Graph showing the levels of *VCAM-1* and *ICAM-1* mRNA as determined by quantitative real-time PCR analysis; *GAPDH* was used as an internal reference gene. **p < 0.01 vs. the control group; ^#^ *p* < 0.05, ^##^ *p* < 0.01 vs. the model group (*n* = 4–6).

AS is known to involve an ongoing inflammatory response, with macrophages and T cells playing key roles in the onset and development of this condition (Hansson and Hermansson, 2011). Immunofluorescence analysis of atherosclerotic lesion areas in aortic arches revealed that surgically-induced menopause resulted in a marked accumulation of CD68^+^ cells (macrophages) and CD3^+^ cells (T cells) (Figure 2A–C). However, inflammatory cell infiltration in the G1 group and both BZBS groups was significantly reduced (Figure 2A–C). The NF-κB pathway serves as a critical mediator of inflammatory responses by activating the transcription of several pro-inflammatory genes (Taylor and Cummins, 2009). We observed that the p-IκBα/IκBα and p-p65/p65 ratios in the aortas of the model group were significantly higher than those of the control group. G1 and BZBS treatment significantly reduced these ratios, bringing them closer to those of the control group (Figures 2D,E). Activation of the NF-κB pathway can lead to the expression of the adhesion proteins VCAM-1 and ICAM-1 (Sun et al., 2020b). The mRNA and protein levels of these adhesion molecules were also assessed. Compared with the control mice, the expression levels of VCAM-1 and ICAM-1 were higher not only in the serum (protein level) but also in the aorta (RNA level) in the high fat diet-fed PM mice. G1 and BZBS reduced the expression of VCAM-1 and ICAM-1 (Figures 2F,G).

### Bazi Bushen Capsule Treatment Inhibited Apoptosis in Ovx/ApoE^-/-^ Mice

**FIGURE 3:**
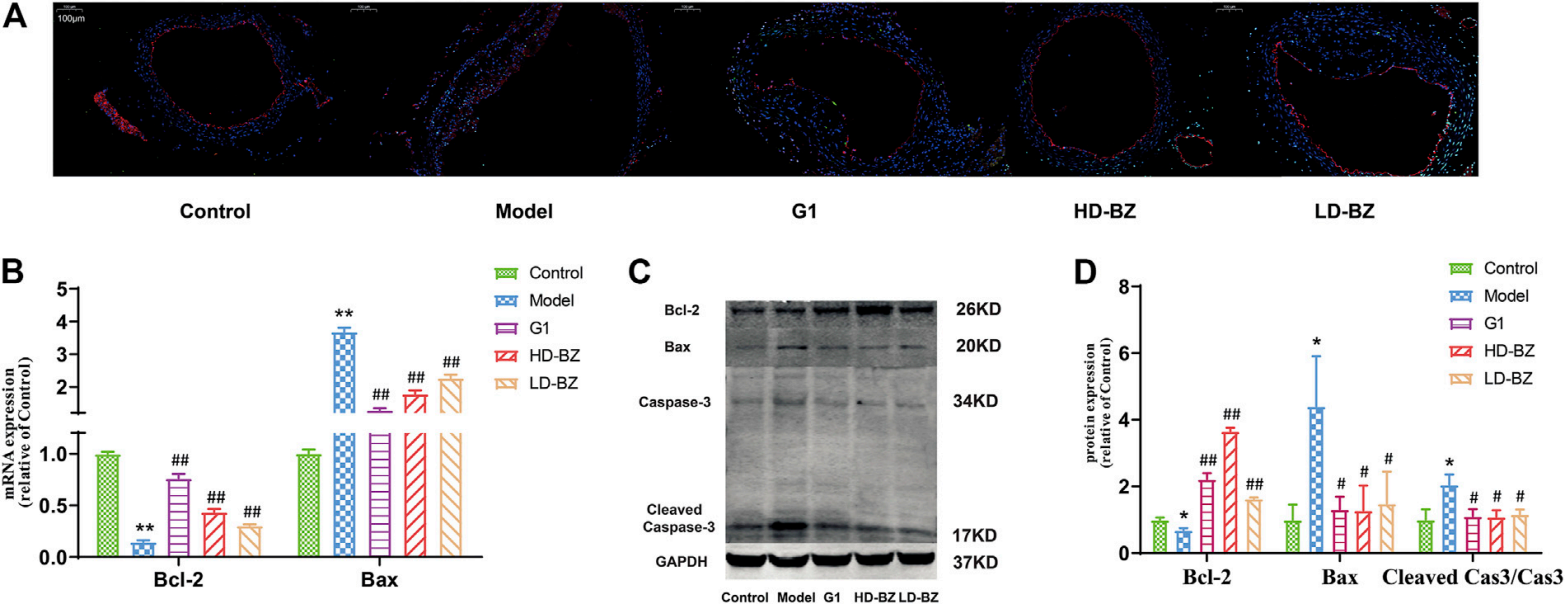
Effect of BZBS on endothelial cell apoptosis in Ovx/ApoE^-/-^mice. **(A)** Images from the TUNEL apoptosis assay were used to quantify apoptotic ECs in aortic arch atherosclerotic plaques in mice. **(B)** *Bcl2* and *Bax* mRNA levels were determined by quantitative real-time PCR analysis. *GAPDH* was used as an internal reference gene. **(C)** Western blots showing Bcl2, Bax, and caspase-3 analytes. **(D)** Graph showing the values derived from the quantification of the bands shown in C; GAPDH was used as an internal reference protein. The data are presented as the mean ± SD. *p < 0.05, **p < 0.01 vs. the control group; ^#^ *p* < 0.05, ^##^ *p* < 0.01 vs. the model group (*n* = 4–6).

Inflammation often causes apoptosis. TUNEL apoptosis assays showed that there were significantly fewer apoptotic ECs in the atherosclerotic plaques of the BZBS groups than in those of the model group (Figure 3A). Model mice had markedly lower expression levels (at both the mRNA and protein levels) of the anti-apoptotic gene, *Bcl-2*, than control mice, whereas the opposite result was observed for the pro-apoptotic gene, *Bax*. However, G1 and BZBS attenuated the effects of ovariectomy on both genes, increasing *Bcl-2* expression and reducing *Bax* expression (Figures 3B–D). We also assessed another apoptotic index, the ratio of cleaved caspase-3 to caspase-3. As shown in Figures 3C,D, mice in the model group had higher ratios than control mice, whereas G1 or BZBS treatment reduced the ratio. In other words, BZBS reduced estrogen deficiency-induced apoptosis by regulating Bcl-2, Bax and cleaved caspase-3.

### G-Protein-Coupled Estrogen Receptor 1 is a Key Mediator of Bazi Bushen Capsule-Induced Endothelial Protection

**FIGURE 4:**
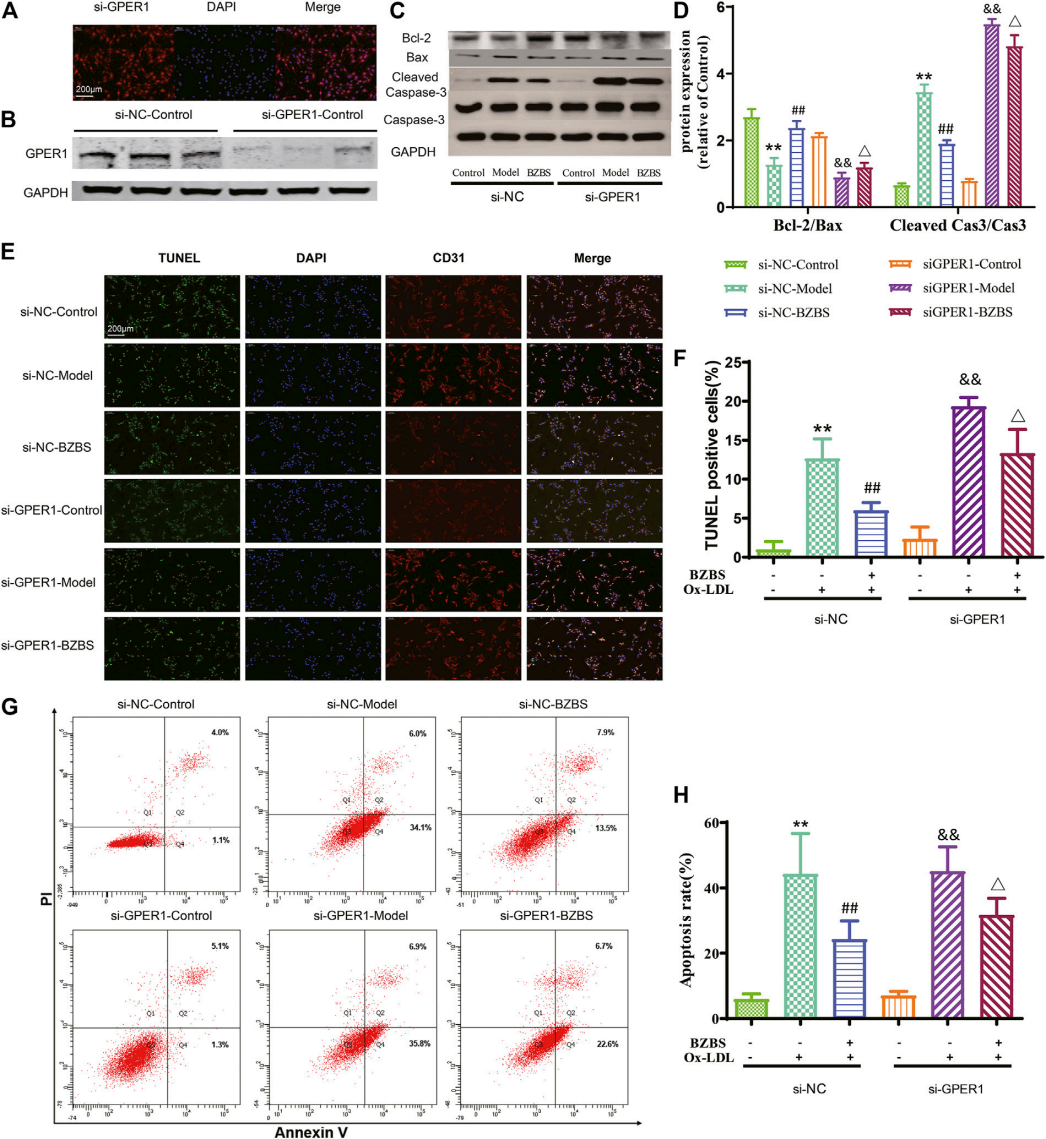
Effect of BZBS on the apoptosis of Ox-LDL-induced HUVECs with GPER1 deficiency. **(A,B)** GPER1 protein levels were measured 40 h posttransfection to evaluate knockdown efficiency. **(C,D)** Western blots showing Bcl2, Bax, and caspase-3 proteins. **(E,F)** Annexin V-FITC/propidium iodide (PI) **(G,H)** was used to measure the apoptotic rates in the various groups of Ox-LDL-treated HUVECs. The data are presented as the mean ± SD. **p < 0.01 vs. the si-NC control group; ^*##*^ *p* < 0.01 vs. the si-NC model group; ^&&^ *p* < 0.01 vs. the si-GPER1 control group; ^*△*^ *p* < 0.05 vs. the si-GPER1 model group (*n* = 3).

Selective *GPER1* activation can inhibit postmenopausal AS (Jing et al., 2017). To determine whether GPER1 mediates the endothelial-protective effect of BZBS, GPER1 expression was knocked down in HUVECs by transfection with GPER1-specific siRNA (Figures 4A,B). Apoptosis was induced with 120 μg/ml Ox-LDL. At this concentration, more than half of the cells remained viable (60.04 ± 3.35%, Supplementary Figure S1A). As shown in Supplementary Figure S1B, BZBS increased cell viability, with the increase being most prominent at 400 μg/ml BZBS. As other kinds of vascular cells such as smooth muscle cells were also included in aortic arch atherosclerotic plaques, Figure 3 does not represent the complete results for endothelial cells. Here a similar result was observed with respect to the effect of BZBS on HUVECs.

After the GPER1 gene was knocked down, Bcl2, Bax, cleaved caspase-3 and caspase-3 were almost unchanged between the si-GPER1-control and si-NC-control groups. The Bcl2/Bax ratio decreased slightly (−20.8%, *p* = 0.088) and the cleaved caspase-3/caspase-3 ratio increased slightly (19.6%, *p* = 0.158) in the si-GPER1-control group, but the differences were not significant. These results indicate that silencing GPER1 in unstimulated HUVECs may slightly induce apoptosis. Interestingly, after stimulation with Ox-LDL, the Bcl2/Bax ratio in both model groups decreased compared with that in each control group, while the cleaved caspase-3/caspase-3 ratio was significantly increased. BZBS improved these ratios to some extent in the si-GPER1-BZBS group but had a reduced effect compared to that in the si-NC-BZBS group (BZBS upregulated Bcl2/Bax 85.9% in the si-NC group vs. 33.2% in the si-GPER1 group and downregulated cleaved caspase-3/caspase-3 44.8% in the si-NC group vs. 12.0% in the si-GPER1 group; Figures 4C,D). TUNEL and Annexin V-FITC/PI assays also showed that the apoptotic rates in the si-GPER1-control groups were generally higher than those in the corresponding si-NC-control groups (Figures 4E–H). Ox-LDL induced more apoptosis in the si-GPER1 group than in the control group, and the anti-apoptotic effect of BZBS was weaker in si-GPER1 cells than in control cells, suggesting that GPER1 plays a key role in protecting ECs (Figures 4E–H). Although there was no significant difference among the groups in early apoptosis (Supplementary Figure S2A), the same result was found in late apoptosis (Supplementary Figure S2B).

## Discussion

The accumulation of lipids in the arterial walls induces AS (Salvayre et al., 2016; Suciu et al., 2018). Sex hormone levels after menopause are associated with increased AS risk in women later in life (Zhao et al., 2018). PEs have been shown to prevent the development of atherosclerotic lesions via lipid metabolism pathways (Chen et al., 2018), and we demonstrated that BZBS contains 11 unique PEs, and improve fatty acid metabolism in high-fat diet-fed, surgically-induced menopausal ApoE^-/-^ mice by reducing serum lipid levels (Huang et al., 2020). Although the mouse model used is quite complex, it also successfully simulated the clinically severe hyperlipidemia state after menopause. This animal model is widely used in the hyperlipidemia model of postmenopausal women (Meng et al., 2021b; Sun et al., 2020a). In this study, we aimed to investigate the potential mechanism of BZBS on AS. Lipid deposition was higher after high-fat diet-feeding and spaying ovariotomy. Treatment with BZBS reduced lipid deposition, and BZBS exhibited a clear effect against atherogenesis *via* GPER1-dependent anti-inflammatory and anti-apoptotic mechanisms, which revealed that BZBS exhibited a protective effect against AS.

**FIGURE 5:**
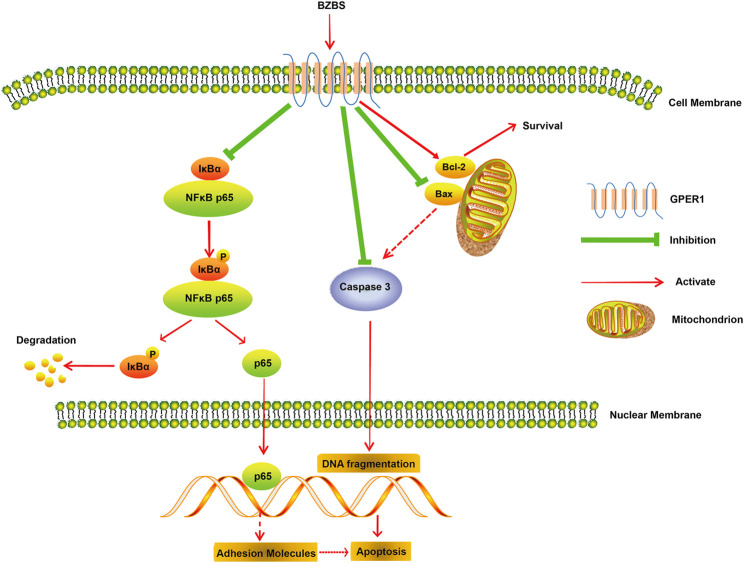
Mechanism by which BZBS inhibits endothelial cell apoptosis through GPER1 mediated regulation.

AS is a chronic inflammatory disease, and lipid metabolism mediates AS-related inflammation (Zhu et al., 2018; Wu et al., 2020). In atherosclerotic lesions, macrophages, T lymphocytes, B-lymphocytes, dendritic cells, ECs, and resident immune cells are activated and accumulate, manifesting inflammatory effects (Pyka-Fosciak et al., 2013). Our immunofluorescence analysis demonstrated that BZBS treatment reduced the accumulation of CD68^+^ macrophages and CD3^+^ T cells. Inflammation-activated ECs release pro-inflammatory mediators, including chemokines, pro-inflammatory cytokines, and adhesion molecules (Song et al., 2016). ECs are the main target of estrogen-mediated vascular protection (Sun et al., 2020a). Estrogen acts as an anti-inflammatory agent by reducing the expression of adhesion proteins via inhibition of the NF-κB pathway (Zhang et al., 2003; Knowlton and Lee, 2012). In this study, the data revealed that BZBS treatment may block the NF-κB signalling pathway, thereby reducing the secretion of adhesion molecules VCAM-1 and ICAM-1 (Figures 2, 5), indicating an anti-inflammatory effect.

EC apoptosis occurs throughout the early stages of AS, and is an important characteristic of this condition (Hort et al., 2014). NF-κB regulates the expression of genes associated with EC apoptosis (Cao et al., 2016). Bcl-2 family members are either anti-apoptotic, such as Bcl-xl and Bcl-2, or pro-apoptotic, such as Bad, Bak, and Bax. The latter proteins are associated with mitochondrial permeability transition pore (MPTP) regulation and the release of cytochrome c (Cyt-c), a critical mitochondrial death factor, resulting in the activation of caspase-9 and caspase-3 and, ultimately, apoptosis (Solesio et al., 2013). Caspase-3 is considered the final executor, as it is indispensable for apoptotic chromatin condensation and DNA fragmentation (Lu et al., 2010). Our data indicated that BZBS markedly inhibited apoptosis, as shown by the upregulation of the anti-apoptotic protein Bcl-2, downregulation of pro-apoptotic Bax, and decreased expression and activity of caspase-3 (Figures 3, 5). This finding was confirmed by immunofluorescence analysis.

**FIGURE 6:**
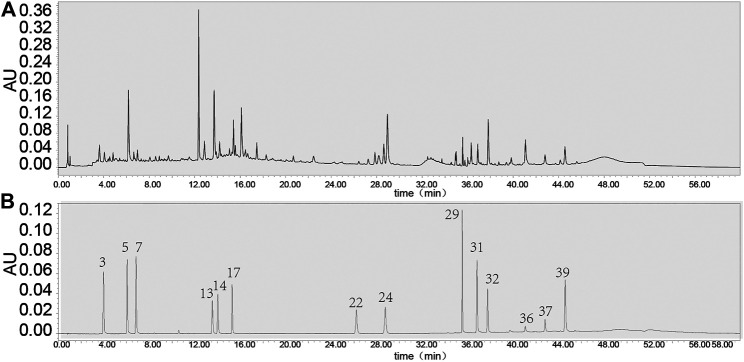
UPLC of BZBS **(A)** and Reference substance [**(B)**, mixed reference substance]. The BZBS detection method is shown as a Supplementary method. Compound 13. Isoquercitrin; compound 14. Hyperoside; compound 17. Verbascoside; compound 22. Epimedins A; compound 24. Icariin; compound 29. Baohuoside I; compound 31. Imperatorin; compound 32. Osthole; compound 36. Catalpol; compound 37. Deoxyschizandrin; 39.Schisandrin B.

As a transmembrane protein, GPER1 contains 375 amino acids and belongs to the G-protein-coupled receptor family. As such, its peptide chain starts with an extracellular N-terminus, traverses the membrane seven times, and ends with an intracellular C-terminus that contains the binding site of a guanosine-binding protein (G protein) (Meyer et al., 2011). Upon binding to estrogen, GPER1 activates several intracellular signal transduction pathways, such as the NF-κB (Du et al., 2018) and caspase-3 signalling pathways (Jiang et al., 2019), decreases the Bax/Bcl-2 ratio (He et al., 2018) and exerts protective effects by inhibiting NF-κB-mediated inflammation and caspase-3- mediated apoptosis. It is well known that PEs exhibit estrogenic activity. The pharmacological effects of some PEs were sensitized by activating GPER1 (Du et al., 2018; Ariyani et al., 2019). In our previous study, 11 unique PEs were identified in BZBS (the relative levels of 11 unique PEs are shown in Table 3, and Figure 6). G1 was selected as a GPER1 agonist because G1 is devoid of uterotrophic activity, similarly to PEs (Meyer et al., 2014). Indeed, our previous study revealed that BZBS did not prevent uterine atrophy in Ovx/ApoE^-/-^ mice (Huang et al., 2020). In Ox-LDL-stimulated HUVECs, the Bcl2/Bax ratio decreased compared with that of the control group, while the cleaved caspase-3/caspase-3 ratio was increased significantly after the GPER1 gene was knocked down (Figures 4, 5). TUNEL and Annexin V-FITC/PI assays also showed increased Ox-LDL-mediated apoptosis in the si-GPER1 group, and the anti-apoptotic effect of BZBS was reduced in si-GPER1 cells, suggesting that BZBS exerted a protective effect on ECs (Figure 5) via GPER1-dependent anti-inflammatory and anti-apoptotic mechanisms. In this study, results showed that BSBZ and G1 (GPER agonist) reduce apoptosis in HUVECs through activated GPER, which is contrary to previous observation that GPER activation stimulates cell apoptosis (Gros et al., 2013; Feldman et al., 2014): we speculate that the *in vivo* situation is likely very different from the *in vitro* observation (Ding et al., 2009). In addition, the research objects of previous observation are vascular smooth muscle cells. The object of this study is vascular endothelial cells. Some literature studies have shown that the effect of E2 and GPER on endothelial cells is to inhibit apoptosis, while is to promote apoptosis on smooth muscle cells (Kassi et al., 2015; Jing et al., 2017; Wei et al., 2020). In our research, we also found that BSBZ and G1 (GPER agonist) reduce the apoptosis in HUVECs through activated GPER.

**TABLE 3:** Precision test of relative area.

Peak	RT (time)	Relative Area					Mean	SD	RSD%
		Test 1	Test 2	Test 3	Test 4	Test 5			
1	3.52	0.40	0.41	0.41	0.40	0.41	0.40	0.00	0.44
2	3.72	0.90	0.91	0.89	0.91	0.91	0.90	0.01	1.09
3	4.15	1.71	1.69	1.65	1.66	1.69	1.68	0.02	1.43
4	4.93	0.40	0.41	0.41	0.41	0.40	0.41	0.00	1.13
5	6.3	6.84	6.91	6.90	6.86	6.84	6.87	0.03	0.45
6	6.8	0.21	0.21	0.21	0.21	0.21	0.21	0.00	1.62
7	7.12	1.33	1.33	1.37	1.31	1.35	1.34	0.02	1.66
8	8.24	0.43	0.42	0.42	0.42	0.42	0.42	0.01	1.49
9	8.74	0.85	0.85	0.85	0.85	0.85	0.85	0.00	0.34
10	9.88	1.26	1.29	1.24	1.26	1.25	1.26	0.02	1.53
11	12.67	6.71	6.62	6.73	6.70	6.68	6.69	0.04	0.59
12	13.16	2.96	2.94	2.96	2.91	2.86	2.92	0.04	1.41
13^a^	14.02	4.91	4.99	4.93	4.96	4.97	4.95	0.03	0.60
14^a^	14.47	1.17	1.16	1.21	1.16	1.16	1.17	0.02	1.88
15	14.73	1.03	1.03	1.04	1.02	1.03	1.03	0.01	0.82
16	15.33	0.45	0.45	0.46	0.45	0.45	0.45	0.00	0.34
17^a^	15.71	1.09	1.11	1.10	1.10	1.10	1.10	0.01	0.56
18	16.43	3.35	3.43	3.35	3.36	3.30	3.36	0.05	1.44
19	16.82	0.42	0.43	0.43	0.43	0.42	0.42	0.00	0.73
20	17.85	2.74	2.76	2.66	2.68	2.72	2.71	0.04	1.54
21	21.2	1.07	1.08	1.12	1.07	1.08	1.08	0.02	1.77
22^a^	27.92	1.75	1.74	1.75	1.72	1.78	1.75	0.02	1.16
23	28.52	6.50	6.54	6.59	6.43	6.50	6.51	0.06	0.94
24^a^	29.31	3.98	3.89	3.85	3.81	3.81	3.87	0.07	1.80
25	29.64	19.31	18.73	18.96	19.40	19.31	19.14	0.29	1.50
26	34.71	0.16	0.17	0.16	0.16	0.16	0.16	0.00	1.89
27	35.07	1.69	1.77	1.72	1.70	1.70	1.72	0.03	1.89
28	35.71	1.32	1.36	1.36	1.34	1.34	1.35	0.02	1.15
29^a^	35.93	0.37	0.37	0.37	0.37	0.36	0.37	0.00	1.28
30	36.5	2.45	2.44	2.51	2.50	2.48	2.47	0.03	1.27
31^a^	37.09	3.15	3.19	3.25	3.21	3.19	3.20	0.03	1.07
32^a^	38.05	6.68	6.74	6.63	6.66	6.73	6.69	0.05	0.73
33	39.02	0.42	0.43	0.42	0.42	0.42	0.42	0.00	1.03
34	39.71	0.66	0.65	0.67	0.66	0.66	0.66	0.01	0.87
35	40.17	2.05	2.09	2.11	2.05	2.04	2.07	0.03	1.36
36^a^	41.49	3.53	3.58	3.49	3.59	3.54	3.55	0.04	1.10
37^a^	43.31	2.55	2.59	2.58	2.51	2.61	2.57	0.04	1.43
38	44.75	0.67	0.65	0.66	0.67	0.66	0.66	0.01	1.54
39^a^	45.18	2.53	2.65	2.64	2.67	2.62	2.62	0.05	2.04

a Phytoestrogens 13. Isoquercitrin; 14. Hyperoside; 17. Verbascoside; 22. Epimedins A; 24. Icariin; 29. Baohuoside Ⅰ; 31. Imperatorin; 32. Osthole; 36. Catalpol; 37. Deoxyschizandrin; 39.Schisandrin B.

However, this study also has some limitations. First, BZBS is a complex molecular mixture, and not all of the contents are completely known. Second, the specific PEs that play major roles have not been determined. Cell membrane chromatography is a very efficient method and was developed to identify bioactive compounds from TCMs (Sun et al., 2020b), this method can be used to characterize the 11 phytoestrogens in BZBS with respect to anti-atherosclerosis effects. Third, GPER1 overexpression was not considered. Fourth, the effects of AS progression through other pathways require further study, such as classic estrogen receptors (ERα, ERβ and several splice variants). We deduced that BZBS can reduce vascular endothelial cell pyroptosis through ERα-mediated activation of autophagy or the following regulation of the expressions of SREBPs and SIRT1 to improve atherosclerosis in post-menopausal stage (Chen, et al., 2020; Meng et al., 2021a). Finally, for extracts already used in humans, it is critical to have a dose–response curve in the experimental set up that includes the usual dose in humans (Heinrich et al., 2020). Current dose usage in our study (1.4 and 2.8 mg/kg/d) was slightly higher than the clinical equivalent dose in mice (0.7 mg/kg/d). Although the dose is not grossly excessive, the lower dose studies will be needed.

## Conclusion

In this study, we revealed that BZBS treatment reduced lipid deposition, inhibited inflammatory molecule expression, protected against apoptosis, and decreased atherosclerotic lesion formation in high-fat diet-fed, surgically-induced menopausal mice. We further indicated that the underlying mechanism of these anti-inflammatory and anti-apoptotic effects was mediated by the GPER1 pathway. Our findings indicate that BZBS may prevent AS in postmenopausal women.

## Data Availability

The original contributions presented in the study are included in the article/Supplementary Material, further inquiries can be directed to the corresponding authors.

## Abbreviations

AS, atherosclerosis; BZBS, bazi bushen capsule; CMC, carboxymethylcellulose; ECs, endothelial cells; GPER1, G-protein-coupled estrogen receptor 1; NF-κB, nuclear factor kappa B; Ovx, ovariectomy; PEs, phytoestrogens; PM, post-menopausal
