# Corrigendum: Bazi Bushen Capsule Alleviates Post-Menopausal Atherosclerosis *via* GPER1-Dependent Anti-Inflammatory and Anti-Apoptotic Effects

**DOI:** 10.3389/fphar.2021.774792

**Published:** 2021-10-04

**Authors:** Dan Huang, Xindong Wang, Yunhong Zhu, Juexiao Gong, Junqing Liang, Yanfei Song, Yiyan Zhang, Linsheng Liu, Cong Wei

**Affiliations:** ^1^ Affiliated Hospital of Integrated Traditional Chinese and Western Medicine, Nanjing University of Chinese Medicine, Nanjing, China; ^2^ Jiangsu Province Academy of Traditional Chinese Medicine, Nanjing, China; ^3^ National Key Laboratory of Collateral Disease Research and Innovative Chinese Medicine, Shijiazhuang, China; ^4^ Department of Clinical Pharmacology, The First Affiliated Hospital of Soochow University, Suzhou, China

**Keywords:** atherosclerosis, bazi bushen capsule, lipid profile, inflammation, GPER1 antagonist, apoptosis

In the original article, there was a mistake in Table 1 as published. BZBS is a post-marketing drug, and there is an error in the components of BZBS and ratio amounts in application (Li et al., 2021). The corrected Table 1 appears below.

**TABLE 1:** The ratio of the plants present in the preparation of BZBS.

Plants	Amount in application (ratio)
*Cuscuta chinensis* Lam. [Convolvulaceae; Cuscutae semen]	250
*Lycium barbarum* L. [Solanaceae; Lycii cortex]	138
*Schisandra chinensis* (Turcz.) Baill. [Schisandraceae; Schisandrae chinensis fructus]	46
*Cnidium monnieri* (L.) Cusson [Apiaceae; Cnidii fructus]	35
*Rosa laevigata* Michx. [Rosaceae; Rosae laevigatae fructus]	35
*Rubus chingii* Hu [Rosaceae; Rubi fructus]	35
*Allium tuberosum* Rottler ex Spreng. [Amaryllidaceae; Allii tuberosi semen]	35
*Toosendan fructus* [Meliaceae; Fuctus Toosendan]	23
*Epimedium brevicornu* Maxim. [ Berberidaceae; Epimedii folium]	70
*Morindae officinalis* radix [Rubiaceae; Radix Morindae Offcinalis]	35
*Cistanche deserticola* Ma [Orobanchaceae; Cistanches herba]	35
*Rehmannia root* [Orobanchaceae; Radix Rehmanniae Recens]	46
*Cyathula officinalis* K.C.Kuan [Amaranthaceae; Cyathulae radix]	35
*Panax ginseng* C.A.Mey. [Araliaceae; Ginseng radix et rhizoma]	25
*Cervus nippon* Temminck [Cervidae; Cervi Cornu Pantotrichum]	16
*Hippocampus Kelloggi* [Syngnathidae; Hippocampus]	21

The authors apologize for this error and state that this does not change the scientific conclusions of the article in any way. The original article has been updated.
