# Supplementary material for: Bazi Bushen Capsule Alleviates Post-Menopausal Atherosclerosis via GPER1-Dependent Anti-Inflammatory and Anti-Apoptotic Effects
Source: Front Pharmacol. 2021 Jun 25;12:658998. doi: 10.3389/fphar.2021.658998 (PMC8267998; doi:10.3389/fphar.2021.658998)
Supplement: Supplementary file 1 [file DataSheet1.DOCX]

**Bazi Bushen** **capsule alleviates post-menopausal atherosclerosis via GPER1-dependent anti-inflammatory and anti-apoptotic effects**

Dan Huang ^1,2,a^ , Xindong Wang ^1,2,a^, Yunhong Zhu^1,2^, Juexiao Gong^1,2^, Junqing Liang^4^, Yanfei Song^4^, Yiyan Zhang^1,2^, Linsheng Liu ^3,^*, Cong Wei ^4,^**

^1^ Affiliated Hospital of Integrated Traditional Chinese and Western Medicine, Nanjing University of Chinese Medicine, Nanjing 210028, China;

^2^Jiangsu Province Academy of Traditional Chinese Medicine, Nanjing 210028, China;

^3^ Department of Clinical Pharmacology, The First Affiliated Hospital of Soochow University, Suzhou 215006, China;

^4^ National Key Laboratory of Collateral Disease Research and Innovative Chinese Medicine, Shijiazhuang 050035, China

^a^ Equally contributed to this work

*Corresponding author. [liulinsheng@suda.edu.cn (L](mailto:liulinsheng@suda.edu.cn%20(L).Liiu)

**Corresponding author. [weicong@yiling.cn (C.](mailto:weicong@yiling.cn%20(C.) Wei)

**Supplementary method**

*Preparation of sample*

Dilute 1 g of Bazi Bushen Capsule sample in 80 mL of 85% methanol, and extract once by ultrasonic for 40 min. After sonication, the sample was centrifuged at 7000 r/min for 5 min, the supernatant was poured into an evaporating dish, and the volume was concentrated to 5mL by evaporation in a water bath, and then filtered with a 0.22μm microporous membrane.

*UPLC condition*

Instrument: Waters UPLC chromatographic analyzer, UV detector. Chromatographic conditions: chromatographic column ACQUITY UPLC BEH C18, 1.7μm with 2.1 * 100mm; Flow rate: 0.3mL/min; full wavelength scan; column temperature: 30℃; injection volume: 1μL; mobile phase: acetonitrile-acid water (0.1% phosphoric acid aqueous solution).

*Immunofluorescence*

Aortic arch sections were analysed using tyramide signal amplification (TSA) with a ZEISS laser scanning confocal microscope (ZEISS Microscopy, Zena, Germany). In brief, after deparaffinize and rehydrate, antigen retrieval with EDTA antigen retrieval buffer (pH 8.0) was executed. Then block endogenous peroxidase with H_2_O_2_ and serum. Incubate slides with the rabbit anti CD68 monoclonal antibody (1:100; Abcam, Cambridge, UK) overnight at 4 ℃, placed in a wet box containing a little water. Corresponding goat anti-rabbit IgG (1:1000; Abcam) marked with HRP, wash slides three times with PBS (pH 7.4) in a Rocker device, 5 min each. Incubate slides with TSA-FITC solution for 10 min in dark condition. After microwave treatment, remove the rabbit polyclonal to CD68 and goat anti-rabbit IgG combined with tissue with EDTA antigen retrieval buffer (pH 8.0). Incubate slides with rabbit polyclonal to CD3 (1:100; Abcam) overnight at 4 ℃, placed in a wet box containing a little water. Wash slides three times with PBS (pH 7.4) in a Rocker device, 5 min each. Then throw away liquid slightly. Cover objective tissue with goat anti-rabbit IgG, incubate at room temperature for 50 min in dark condition. Add TSA-CY3 solution. Incubate with DAPI solution at room temperature for 10 min,kept in dark place. Microscopy detection and collect images by Fluorescent Microscopy using a ZEISS laser scanning confocal microscope.

*Quantitative real-time PCR analysis*

Converted to cDNA with a cDNA Reverse Transcription Kit (Takara Bio Inc., Kusatsu, Japan) and [RNeasy](https://www.promega.com.cn/Resources/Protocols/Technical%20Manuals/Eastep%20Protocols/RNA%20Extraction%20Kit%20LS1040/) Fibrous Tissue Mini Kit (QIAGEN, Hilden, Germany) were chosen to extract total mouse aorta RNA. Quantitative real-time PCR analysis was performed using TB Green Premix Ex Taq II (Tli RNase H Plus) (Takara Bio Inc.) using an iCycler detection system. The expression levels of each gene were determined with the 2^-ΔΔCt^ method using GAPDH as an internal reference gene. The PCR primers are shown in **Table S1**.

*Western blot analysis*

Proteins were extracted with the Minute Total Protein Extraction Kit for Blood Vessels (Invent Biotechnologies, Eden Prairie, MN, USA) according to the manufacturer's protocol. Protein concentrations were determined with a BCA protein assay kit (Beyotime Biotechnology, Shanghai, China).

In each well, 30 μg of protein was loaded and separated by electrophoresis at 140 V in precast mini 12% polyacrylamide gels (GenScript Biotech Corp., Nanjing, China). Proteins were then semidried and transferred onto nitrocellulose membranes (Millipore Corporation, Billerica, MA, USA). After 1 h at 37°C in a blocking solution (LI-COR Biosciences, Lincoln, NE, USA), membranes were incubated overnight at 4°C with monoclonal or polyclonal antibodies. Primary antibodies against IκBα, phospho-IκBα (Ser36), ICAM-1, VCAM-1, Bax, Bcl-2, and Caspase-3 were purchased from Abcam. Antibodies against NF-κB p65, phospho-NF-kB p65 (Ser536), and GAPDH were purchased from Cell Signaling Technology (CST) (Danvers, MA, USA). The following day, the membranes were incubated with a goat anti-mouse/rabbit IgG secondary antibody at room temperature for 1 h. The Odyssey imaging system and image analysis software (LI-COR Biosciences) were used to quantify the amount of target protein by grey scanning. GAPDH was used as an internal reference protein (**Table S2**).

*Annexin V-FITC/propidium iodide (PI) apoptosis assay*

Annexin V-FITC/propidium iodide (PI) staining was performed with the Invitrogen Annexin V-FITC Apoptosis Detection kit (Thermo Fisher Scientific). Treated cells were measured using a flow cytometer.

Table S1 Primer sequences used for gene expression determination.

| Mice Gene | Primer sequences |
| --- | --- |
| VCAM-1 | Forward :ATTTTCTGGGGCAGGAAGTT |
|  | Reverse: ACGTCAGAACAACCGAATCC |
| ICAM-1 | Forward: GTGATGCTCAGGTATCCATCCA |
|  | Reverse: CACAGTTCTCAA AGCACAGCG |
| Bcl2 | Forward: CAGGTATGCACCCAGAGTGA |
|  | Reverse: GTCTCTGAAGACGCTGCTCA |
| Bax | Forward: GTGAGCGGCTGCTTGTCT |
|  | Reverse: GGTCCCGAAGTAGGAGAGGA |
| GAPDH | Forward: CTACAGCAACAGGGTGG |
|  | Reverse: TATGGGGGTCTGGGATGG |

Table S2 Primary Antibodies

|  | Antibody | Lot. No. | Dilution |
| --- | --- | --- | --- |
| Primary Antibody | CD3 | ab5690 | 0.111111 |
|  | CD68 | ab125212 | 0.111111 |
|  | CD31 | ab124432 | 0.111111 |
|  | Phospho-NF-kB p65 (Ser536) | CST#3033 | 0.736111 |
|  | NF-κB p65 | CST#8242 | 0.736111 |
|  | phospho-IKBα(S36) | ab133462 | 0.736111 |
|  | IKBα | ab76429 | 0.736111 |
|  | VCAM-1 | ab134047 | 0.736111 |
|  | ICAM-1 | ab171123 | 0.736111 |
|  | Bcl-2 | ab59348 | 0.736111 |
|  | Bax | ab32503 | 0.736111 |
|  | Caspase-3 | ab13847 | 0.388889 |
|  | GAPDH | CST#2118 | 0.736111 |


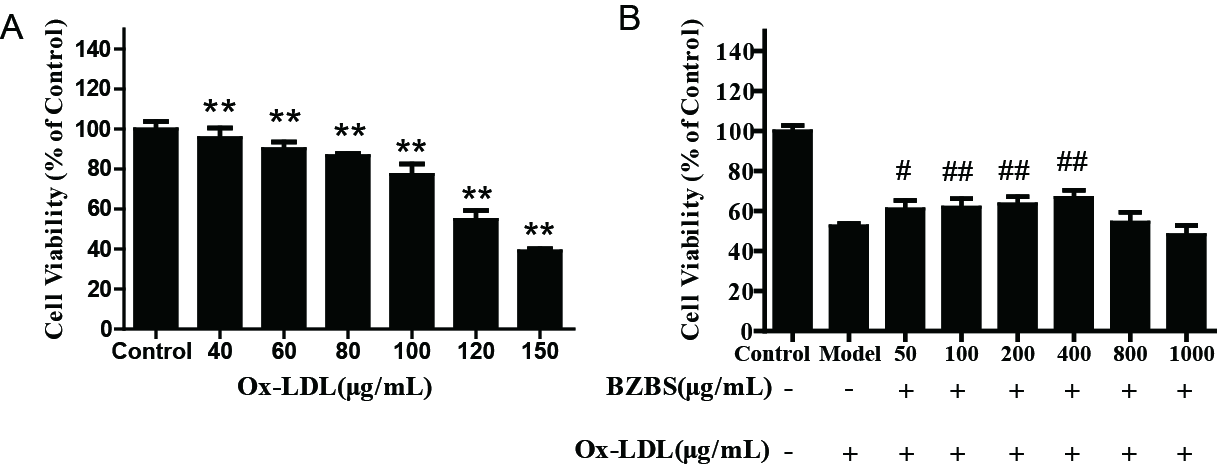


**Fig S1** . (A) Viability of HUVECs treated with various concentrations of Ox-LDL, as assessed by MTS. (B) Viability of HUVECs treated with 120 µg/mL Ox-LDL and various concentrations of BZBS, as assessed by MTS.


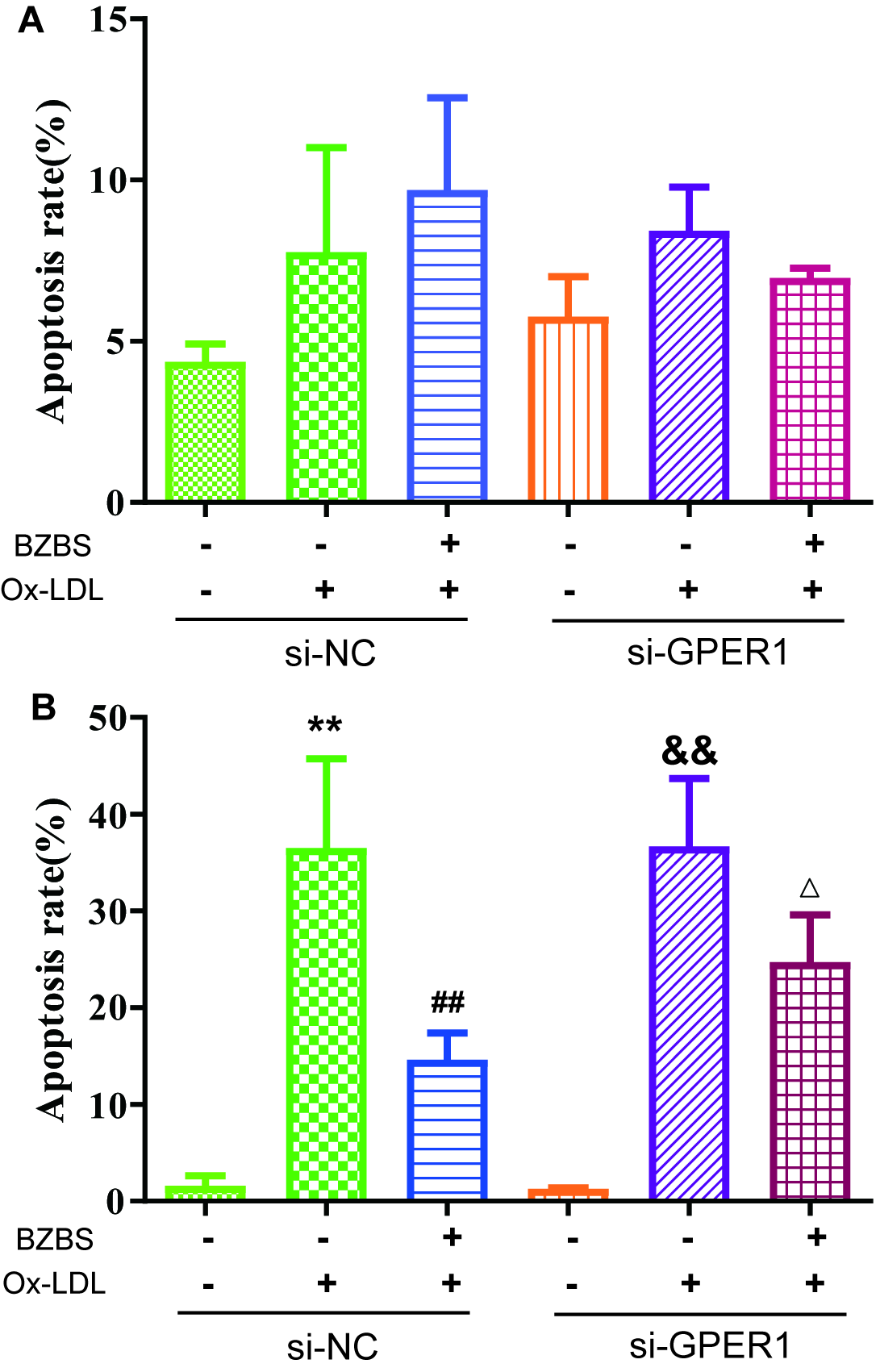


**Fig. S2.** Effect of BZBS on the apoptosis of Ox-LDL-induced HUVECs with GPER1 deficiency.

Annexin V-FITC/propidium iodide (PI) (G,H) was used to measure the apoptotic rates in the various groups of Ox-LDL-treated HUVECs.(A, early apoptosis, B, late apoptosis) The data are presented as the mean ± SD. ^**^*p* < 0.01 vs. the si-NC control group; *^##^p* < 0.01 vs. the si-NC model group; ^&&^*p* < 0.01 vs. the si-GPER1 control group; *^△^p* < 0.05 vs. the si-GPER1 model group (n = 3).
